# Clinical Manifestations and Treatment Outcomes of Syphilitic Uveitis in a Chinese Population

**DOI:** 10.1155/2016/2797028

**Published:** 2016-04-10

**Authors:** Rui Zhang, Jiang Qian, Jie Guo, Yifei Yuan, Kang Xue, Han Yue, Ling Chen

**Affiliations:** ^1^Department of Ophthalmology, Eye and ENT Hospital, Fudan University, Shanghai, China; ^2^Key Laboratory of Visual Impairment and Restoration, Shanghai 200031, China

## Abstract

*Purpose*. To describe the clinical manifestations and treatment outcomes of syphilitic uveitis in a Chinese population.* Methods*. This is a retrospective case series of 15 consecutive patients with syphilitic uveitis treated at a uveitis referral center between 2012 and 2015.* Results*. Fifteen patients were diagnosed with syphilitic uveitis based on positive serological tests. Nine patients were male. Coinfection with human immunodeficiency virus was detected in two patients. Twenty eyes presented with panuveitis and all patients had posterior involvement. The most frequent manifestations were retinal vasculitis and papillitis, while syphilitic posterior placoid chorioretinitis was only found in three eyes. All patients received systemic penicillin therapy according to CDC guidelines. Nine patients were misdiagnosed before presenting to our center and the delay in treatment with penicillin was associated with poor final visual outcomes (*P* < 0.05).* Conclusions*. In our series, both male and female were almost equally affected and coinfection of syphilis with human immunodeficiency virus was uncommon. All patients in this study had posterior involvement and the most common manifestations were retinal vasculitis and papillitis. Syphilis should be considered as an important differential diagnosis especially for posterior uveitis and panuveitis. Early diagnosis and appropriate treatment are important for visual prognosis.

## 1. Introduction

Acquired syphilis is a sexually transmitted disease caused by spirochetal bacterium* Treponema pallidum*. It affects most organ systems including skin, heart and blood vessels, bone, nervous system, and eye [1, 2]. Syphilitic uveitis can occur at any stage of the disease [3]. The ocular manifestations of acquired syphilis are protean and syphilitic uveitis may be included in the differential diagnosis of any form of ocular inflammation [4]. A classic treatment regimen for neurosyphilis with intravenous penicillin G has been considered successful in the treatment of syphilitic uveitis and resulting in good prognosis [5].

The prevalence of syphilis in China has increased rapidly in recent years [6]; however most reports in the literature on clinical features of syphilitic uveitis were from Europe and the United States and only few reports were in Chinese patients. In this study, we investigated the different manifestations and treatment effects of syphilitic uveitis in Chinese patients.

## 2. Materials and Methods

This is a retrospective case series of fifteen consecutive patients with syphilitic uveitis presenting at Eye and ENT Hospital, Fudan University, China, between May 2012 and April 2015. This study was approved by the Hospital Ethics Committee and all processes were in agreement with the Declaration of Helsinki. Written informed consent was obtained from the patients before collection of blood samples.

The diagnosis of syphilitic uveitis was confirmed by positive serologic tests, including rapid plasma regain titer (RPR) and treponema pallidum particle agglutination assay (TPPA) [7]. The International Uveitis Study Group criteria were used for the classification of uveitis [8]. Anterior chamber (AC) cells and flare were graded on the ordinal scales and vitreous cells and haze were graded based on standard photographs developed by Nussenblatt and associates, with the modification adopted by the SUN Working Group [8, 9]. The gradings of vitreous cells, vitreous haze, and location of inflammation were made with the pupil dilated [10]. Each patient underwent complete ophthalmologic examination including best-corrected visual acuity (BCVA), slit-lamp biomicroscopy, applanation tonometry, ophthalmoscopy, and B-scan ultrasonography. Color and fundus fluorescein angiography (FFA) were obtained in each case except for patients whose fundus were blurred with dense vitritis. Optical coherence tomography (OCT), electroretinogram (ERG), and visual evoked potential (VEP) were performed in selected patients. Concomitant systemic findings were collected including the presence of mucocutaneous lesions and human immunodeficiency virus (HIV) antibody status.

All patients received the standard treatment for neurosyphilis, intravenous penicillin G at the dose of 18–24 million units per day for 2 weeks [11], or an alternative regimen of intravenous ceftriaxone at a dose of 2 g per day for 2 weeks for those who are allergic to penicillin [4]. Topical corticosteroid and cycloplegic drops were used in patients with anterior chamber inflammation. All patients were followed by the same uveitis specialist and the follow-up time ranged from 6 months to 23 months. Any changes of ocular inflammation and visual acuity were recorded. At the end of treatment, ophthalmologic examination and the laboratory evaluation for syphilis were repeated. The treatment was considered successful if the patients had no ocular inflammation in both eyes and serologic test of RPR was negative after completion of therapy.

## 3. Results

Fifteen patients with syphilitic uveitis were evidenced by ocular inflammation, positive RPR, and TPPA tests. Nine patients (60%) were male and six patients (40%) were female. The median age at presentation was 50 years (range, 35–68 years). Other causes of uveitis were excluded. Serum RPR titers and TPPA were positive in all patients (100%) at presentation. RPR titers ranged from 1 : 8 to 1 : 256 and TPPA were positive (1 : 80). Coinfection with human immunodeficiency virus was detected in two male patients (13.3%) and one of them was homosexual.

**Figure 1:**
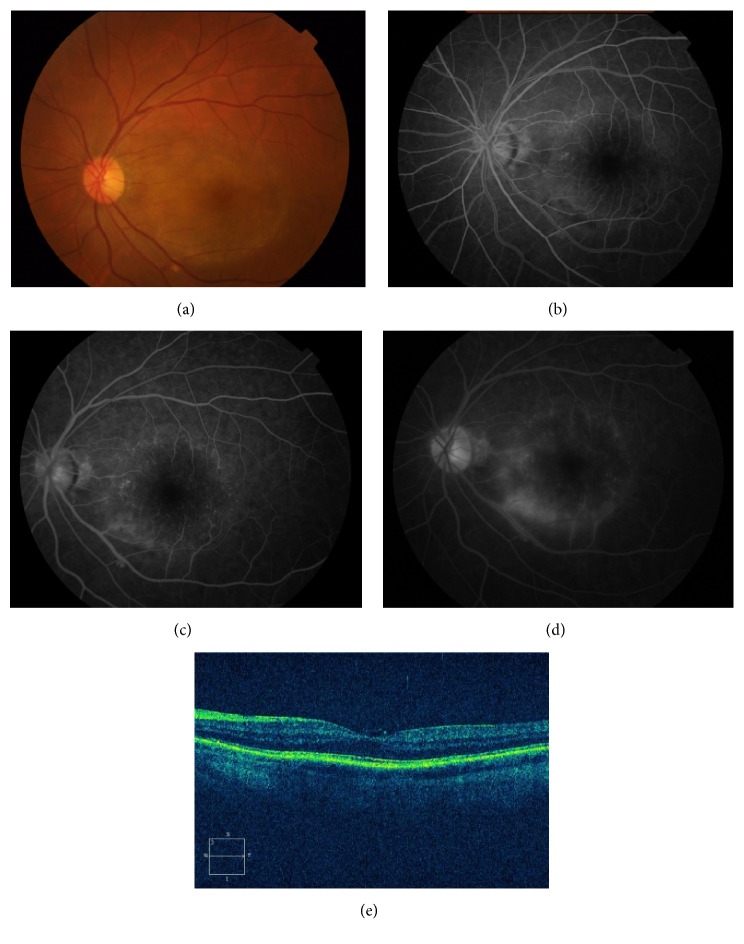
Patient 1 in the tables with acute syphilitic posterior placoid chorioretinitis. (a) Fundus photograph of the left eye showing a placoid, yellowish, outer retinal lesion. (b) Early-phase fluorescein angiogram showing faint hyperfluorescence in the area corresponding to the lesion. ((c) and (d)) Midphase fluorescein angiogram showing progressive hyperfluorescence followed by late staining. (e) OCT scan illustrating partial ill-defined IS/OS junction, irregular RPE, and a fine epiretinal membrane on the retinal surface.

**Figure 2:**
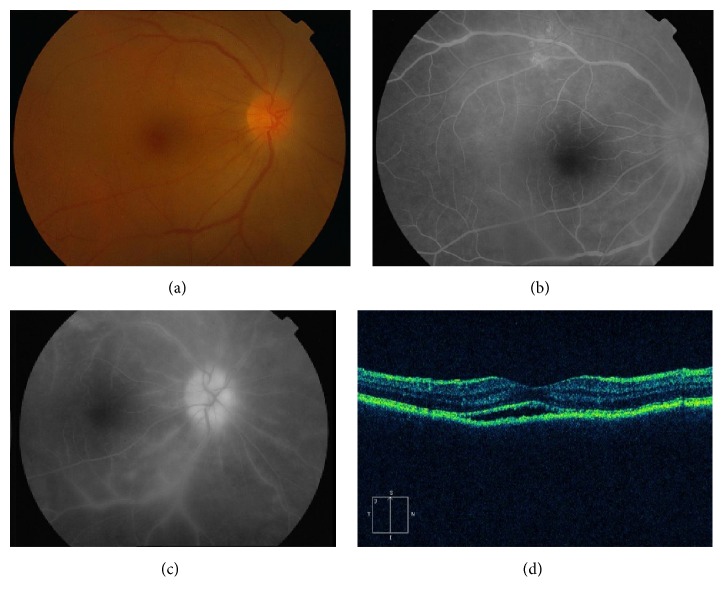
Patient 11 in the tables with syphilitic retinal vasculitis and papillitis. (a) Fundus photograph of the right eye showing disc and retinal edema. ((b) and (c)) Fluorescein angiogram showing hyperfluorescence with leakage of dye from retinal vessels as well as from optic nerve head. (d) OCT scan illustrating subretinal fluid and irregular RPE.

Of the fifteen patients, three (20%) had a history of oral ulcers, two (13.3%) had chancre, one (6.7%) suffered from headache, two (13.3%) had genital ulcers, and two (13.3%) had skin rash of secondary syphilis. The follow-up time ranged from 6 months to 23 months with a mean of 10.1 months (Table 1). The duration of ocular symptoms before presentation ranged from 1 month to 18 months and the patients presented with active inflammation or chronic processes. Ocular involvement was bilateral in 11 patients (73.3%) and unilateral in 4 patients (26.7%). The main complaints were blurry vision in 15 patients (100%), redness in 7 patients (46.7%), floaters in 6 patients (40%), and ocular pain in 3 patients (20%). Clinical manifestations of the involved eyes are presented in Table 2. The initial VA in the 26 affected eyes ranged from 20/80 to hand movement. Twenty eyes (76.9%) had concomitant anterior chamber inflammation and mutton-fat keratic precipitates. Iris involvement may manifest as posterior synechiae in five eyes (19.2%) and iris nodules (Busacca nodules within the iris stroma) in three eyes (11.5%). Eight eyes (30.8%) developed secondary cataract and five eyes (19.2%) had raised intraocular pressure (IOP). Mild-to-severe vitreous opacities were observed in twenty-four eyes (92.3%). Three eyes (11.5%) presented with posterior placoid chorioretinitis, with circular, yellowish, outer retinal lesion (Figure 1). Two eyes (7.7%) had diffused chorioretinitis. Fifteen eyes (57.7%) had retinitis and retinal vasculitis (Figure 2), and papillitis was evident in nine eyes (34.6%). Four eyes (15.4%) had cystoid macular edema (CME) and three (11.5%) had epiretinal membrane (ERM). Serous retinal detachment and retinal splinter hemorrhage were seen in one eye (3.8%), respectively. One patient had pallor optic discs in both eyes (7.7%). The fundus was blurred in six eyes (23.1%) due to vitreous opacity, and retinal edema was detected in those eyes using B-scan ultrasonography.

Signs and symptoms of all patients improved with systemic therapy for syphilis. After treatment, inflammatory cells in anterior chamber and vitreous body decreased and vision improved in all eyes (Table 2). Best-corrected visual acuity at final visit ranged from 20/20 to 20/60, with a median of 20/32. Raised intraocular IOP was controlled medically. No patients were found to increase in the severity of uveitis following a Jarische Herxheimer reaction to treatment. Nine of the fifteen patients were diagnosed previously as other types of uveitis that led to a delay in treatment, and long-standing cystoid macular edema and optic neuropathy resulted in poor visual acuity (*P* < 0.05). RPR titer was negative after completion of therapy in all patients. Systemic manifestations were improved simultaneously. One patient relapsed after treatment and presented with a recurrence of chorioretinitis and concomitant anterior chamber inflammation during the follow-up. She received a new treatment cycle of intravenous penicillin G with topical corticosteroids and cycloplegic drops, and her final visual acuity was 20/25 in the right eye and 20/20 in the left eye, respectively.

## 4. Discussion

Syphilis has reemerged in China and the prevalence of syphilitic uveitis has increased markedly in the past few years [5, 12]. However most reports in the literature on clinical features of syphilitic uveitis were from Europe and the United States and only few reports were from China. In this study, we summarized the clinically distinct features of syphilitic uveitis in Chinese patients. Our data showed both male and female were almost equally affected and coinfection of syphilis with human immunodeficiency virus was uncommon. All patients had posterior involvement and isolated anterior uveitis was rare. Misdiagnosis was common and the delay in treatment was associated with poor final visual outcomes.

Although other series suggested that males were predominantly affected with syphilitic uveitis [4, 13–15], our data showed both male and female were almost equally affected. Coinfection with HIV was also low in our cohort, compared to what reported in the literature. The bias could be partially explained by different race and ethnicity. Vitreous cells were present in the majority of our patients, and involvements of retina and/or choroid were found in all eyes. Isolated anterior involvement was not found in our cohort; in contrast, in a Singaporean population anterior uveitis was reported to be one of the most common manifestations of syphilitic intraocular inflammation [14]. The difference could be partially due to frequent use of FFA in our series, as FFA can help to identify vasculitis, which may not be visualized by fundus exam.

The most common manifestations were retinal vasculitis and papillitis, which were in accordance with previous studies [4]. Acute syphilitic posterior placoid chorioretinitis (ASPPC) was previously reported as a distinctive ocular manifestation of syphilis infection [2, 16, 17] and was identified in a few cases in our series. ASPPC has been postulated to be the result of an active inflammatory reaction at the level of the choriocapillaris-pigment epithelial-retinal photoreceptor complex [2]. Eyes with ASPPC typically presented as yellow-white, placoid, circular, or oval lesion in the macular or extramacular area at the level of RPE (Figure 1(a)). Fundus fluorescein angiography revealed early-phase hypofluorescent or faint hyperfluorescent central lesion and staining in the later frames (Figures 1(b), 1(c), and 1(d)). Although the incidence of HIV coinfection was previously reported higher in patients with ASPPC (nearly 40%) [16], none of the patients with ASPPC in our cohort were found to be HIV positive. Vitreous opacity was another important sign in Chinese patients suffering from syphilitic uveitis. The opacities could be mild or severe, and multiple scattered preretinal vitreous opacities were characteristic, which can be recognized to assist early diagnosis.

Syphilitic uveitis is one of the few ocular entities that can be cured with appropriate antimicrobial therapy. As the eye is an extension of the CNS, ocular syphilis should be treated as neurosyphilis [11], and the classic treatment regimen for neurosyphilis was intravenous penicillin G [6]. In our study, when a course of penicillin G was completed, ocular inflammation decreased in all patients and best-corrected visual acuity improved significantly. Severe ocular inflammation such as vasculitis or dense vitritis or papillitis may not lead to permanent visual impairment when appropriately treated. The patients with prompt treatment had complete functional and morphological recovery with good visual acuity and normal fundus appearance at final visit. Nine patients in our study were misdiagnosed and treated as noninfectious uveitis prior to visiting our center, and the delay in diagnosis and treatment led to long-standing cystoid macular edema and optic neuropathy, which associated with poor visual acuity. Therefore, early diagnosis and prompt treatment of syphilis are important and any delay may increase the risk of severe ocular complications and irreversible visual loss.

Syphilis uveitis is one of the so-called masquerade syndromes in its ability to mimic various diseases such as atypical presentations of Vogt-Koyanagi-Harada disease, viral retinitis, sarcoidosis, tuberculosis, and intraocular lymphoma [18]. Although the presentation may be various in different patients, certain features were characteristic for syphilitic uveitis, such as posterior placoid chorioretinitis and dense preretinal vitreous opacity. In addition, seven patients in our study had a history of mucocutaneous manifestation of syphilis, which was useful in differentiating this condition clinically from other types of uveitis [19]. Sixty percent of the patients in our cohort were misdiagnosed, which was much higher than previously reported [3]. It is necessary to reemphasize the importance to include syphilis uveitis as differential diagnosis for any form of ocular inflammations, especially posterior uveitis and pan-uveitis.

## 5. Conclusions

Syphilitic uveitis is an important clinical entity in China and various presentations may make early diagnosis difficult. In our cohort, both male and female were almost equally affected, and coinfection of syphilis with human immunodeficiency virus was uncommon. All patients in this study had posterior involvement and the most common manifestations were retinal vasculitis and papillitis, while isolated anterior uveitis was rare. Characteristic acute posterior placoid chorioretinitis and preretinal vitreous opacities were identified in a few cases. Associated systemic involvement consisting of headache and mucocutaneous manifestations may also occur. Misdiagnosis was common, and these clinical features can be recognized to assist early diagnosis. Timely diagnosis and appropriate treatment are crucial for visual prognosis and any delay in treatment of syphilitic uveitis was associated with poor visual prognosis.

## Figures and Tables

**Table 1:** General characteristics of the patients with syphilitic uveitis.

Case	Age/gender	HIV Status	Syphilis serology	Systemic clinical findings	Follow-up time
1	50/M	Negative	RPR+ (1 : 32), TPPA+ (1 : 80)	Skin rash	6 months
2	63/M	Negative	RPR+ (1 : 128), TPPA+	Oral ulcers	8 months
3	55/F	Negative	RPR+ (1 : 64), TPPA+	—	15 months
4	47/M	Negative	RPR+ (1 : 32), TPPA+	Chancre	6 months
5	63/F	Negative	RPR+ (1 : 128), TPPA+	Headache	18 months
6	68/M	Negative	RPR+ (1 : 256), TPPA+	Skin rash and genital ulcers	9 months
7	49/M	Negative	RPR+ (1 : 32), TPPA+	—	23 months
8	55/M	Negative	RPR+ (1 : 8), TPPA+ ESR+	Chancre	11 months
9	57/F	Negative	RPR+ (1 : 16), TPPA+	—	9 months
10	38/F	Negative	RPR+ (1 : 64), TPPA+ ESR+	—	8 months
11	41/F	Negative	RPR+ (1 : 64), TPPA+	—	7 months
12	52/M	Negative	RPR+ (1 : 32), TPPA+	Genital and oral ulcers	8 months
13	46/F	Negative	RPR+ (1 : 256), TPPA+ ESR+	—	10 months
14	35/M	Positive	RPR+ (1 : 64), TPPA+	Oral ulcers	6 months
15	42/M	Positive	RPR+ (1 : 64), TPPA+	—	7 months

ESR: erythrocyte sedimentation rate; F: female; HIV: human immunodeficiency virus; M: male; RPR: rapid plasma regain; TPPA: treponema pallidum particle agglutination.

**Table 2:** Clinical features of the patients with syphilitic uveitis.

Case	Duration (month)	Ocular findings	FA findings	Other auxiliary examinations	Initial BCVA	BCVA at final visit
1	OS: 1	OS: AC cells 1+; flare 1+; vitreous cells 1+; placoid, yellowish, outer retinal lesions	OS: early irregular hyperfluorescence; progressive hyperfluorescence; late staining	OCT: partial ill-defined IS/OS junction; irregular RPE; a fine epiretinal membrane	OS: 20/200	OS: 20/25

2	OD: 6	OD: flare 1+; cataract; vitreous cells 1+; retinal edema and exudates	OD: perivascular leakage; hyperfluorescence; late staining	OCT: ill-defined IS/OS junction; increased central foveal thickening	OD: 20/200	OD: 20/30

3	OU: 8	OD: AC cells 2+; flare 2+; cataract; posterior synechiae; Busacca nodules; vitreous cells 3+; fundus blurred; OS: AC cells 1+; flare 2+; cataract; posterior synechiae; Busacca nodules; vitreous cells 3+; fundus blurred	ND	B-scan: diffuse, high reflectivity in vitreous body; retinal edema	OD: HM OS: CF	OD: 20/50 OS: 20/40

4	OD: 5 OS: 6	OU: AC cells 1+; flare 1+; vitreous cells 2+; disk edema; peripheral infiltrates	OU: optic disk leakage; perivascular staining	OCT: OU: optic disk edema; increased thickening of retina	OD: 20/100 OS:20/125	OD: 20/20 OS: 20/25

5	OD: 5 OS: 3	OD: AC cells 1+; flare 1+; vitreous cells 1+; retinal edema and yellow macular exudative lesion; OS: AC cells 1+; flare 1+; vitreous cells 1+; retinal edema	OD: early irregular hyperfluorescence; late diffuse staining of the lesion OS: optic disk leakage and perivascular staining	OCT: OD: irregular retinal contour; ill-defined IS/OS junction; absent external limiting membrane; OS: optic disk edema; retina edema	OD: 20/200 OS: 20/100	OD: 20/25 OS: 20/20

6	OD: 1	OD: AC cells 1+; flare 1+; vitreous cells 2+; diffuse retinal edema; serous retinal detachment	OD: multifocal areas of leakage; late staining	B-scan: medium reflectivity in vitreous body; retinal detachment	OD: CF	OD: 20/32

7	OD: 2 OS: 15	OD: AC cells 1+; flare 1+; vitreous cells 1+; yellow-white macular lesion and proliferative membrane OS: AC cells 1+; flare 1+; vitreous cells 1+; retinal edema; exudation; yellow-white lesions at the level of RPE	OU: early hypofluorescence; late staining	OD: subretinal fluid; fine membrane on the retinal surface OS: subretinal fluid	OD: 20/200 OS: 20/200	OD: 20/40 OS: 20/50

8	OD: 1 OS: 2	OU: AC cells 1+; flare 2+; vitreous cells 3+, with yellow-white spots; fundus blurred	ND	B-scan ultrasonography: diffuse, medium to high reflectivity in vitreous body; retinal edema	OU: HM	OD: 20/25 OS: 20/32

9	OD: 6	OD: AC cells 1+; flare 1+; vitreous cells 1+; retinal splinter hemorrhage; subretinal exudation	OD: early irregular hyperfluorescence; late staining	ND	OD: 20/80	OD: 20/25

10	OD: 7 OS: 10	OD: vitreous cells 1+; retinal edema; ERM; pigmented spots OS: vitreous cells 1+; retinal edema	OU: retinal vascular leakage; late staining; CME	OCT: OD: increased thickening of neurosensory retina; ERM; CME OS: increased thickening of neurosensory retina; CME	OD: 20/80 OS: 20/125	OD: 20/32 OS: 20/50

11	OU: 2	OU: AC cells 1+; flare 1+; vitreous cells 1+; hyperemic optic disk and edema; diffuse retinal edema	OU: optic disk leakage; retinal vascular leakage; late staining	OCT: OU: increased thickening of neurosensory retina; subretinal fluid and irregular RPE	OD: 20/100 OS: 20/200	OD: 20/20 OS: 20/20

12	OD: 3 OS: 7	OD: AC cells 1+; flare 1+; vitreous cells 2+ with yellow spots; retinal edema; yellow macular lesion OS: AC cells 1+; flare 1+; cataract; posterior synechiae; Busacca nodules; vitreous cells 2+ with yellow spots; retinal edema; CME; ERM	OD: retinal vascular leakage; macular leakage under neurosensory elevation OS: retinal vascular leakage; disc staining; CME; ERM	VEP: amplitude of P100 marked reduction	OD: 20/125 OS: 20/400	OD: 20/32 OS: 20/50

13	OD: 18 OS: 17	OU: cataract; pallor optic disk; retinal vascular sheathing	OU: optic disk staining; perivascular leakage; late staining	Flash and pattern ERG and VEP reduced OU	OD: 20/400 OS: 20/250	OD: 20/40 OS: 20/25

14	OD: 5 OS: 3	OD: cataract; posterior synechiae vitreous cells 1+; midperipheral yellow lesion; CME OS: AC cells 1+; flare 1+; vitreous cells 1+; optic disk edema; retinal edema	OD: early hypofluorescence; late staining; CME OS: optic disk and retinal vascular leakage; late staining	ND	OD: 20/125 OS: 20/100	OD: 20/40 OS: 20/25

15	OU: 2	OD: AC cells 2+; flare 1+; cataract; posterior synechiae; vitreous cells 3+ with yellow spots; fundus blurred OS: AC cells 1+; flare 1+; vitreous cells 3+ with yellow spots; fundus blurred	ND	B-scan ultrasonography: high reflectivity in vitreous cavity with retinal edema	OD: CF OS: 20/500	OD: 20/60 OS: 20/50

AC: anterior chamber; BCVA: best corrected visual acuity; CF: counting fingers; CME: cystoid macular edema; ERG: electroretinogram; ERM: epiretinal membrane; FA: fluorescein angiography; HM: hand movement; IS/OS: inner segment/outer segment; ND: not done; OCT: optical coherence tomography; OD: right eye; OS: left eye; OU: both eyes; VEP: visual evoked potential.
